# CABG in high risk patients: does the type of cardioplegia affect outcomes?

**DOI:** 10.1186/1749-8090-8-S1-O195

**Published:** 2013-09-11

**Authors:** D Pena, D Andrade, F Nunez, H Santos, H Orjuela, V Caicedo

**Affiliations:** 1 Cardiac Surgery, Fundacion Clinica Shaio, Bogota, Colombia

## Background

Several cardioplegic solutions have been previously studied in patients undergoing CABG. However data on elderly high risk surgical patients is lacking. The main objective is to determined differences on mortality and clinical outcomes regarding the type of cardioplegic solution.

## Methods

Consecutive high risk patients with a recient acute coronary syndrome(ACS) undergoing CABG on CPB inducing cardiac arrest were enrolled prospectively. A Comparison between those who received plegisol®(HCS) vs. Custodiol®(HTK) was performed. Statistical analysis of preoperative, operative and postoperative variables were done utilizing spss version 19.0.

## Results

During 36 months, 721 patients underwent CABG. Only patients older that 70 years-old with an acute coronary syndrome were included. High risk was defined as Euroscore I greater than 7. Exclusion criterias were Chronic Renal Failure, off-pump surgery and additional procedures. 85(12%) patients were selected for analysis. Mean Age was 75(70-87). 56(65.9%) were male and 29(34.1%) were female. Mean Euroscore I was 10(8-20). Mean EF was 46%(+/-11). Left-main coronary obstruction was present in 13(15%). Cardioplegic solution was administered randomly. HCS was administered to 40(47%) and HTK to 45(53%) patients. 30 days-Mortality occurred in 7(8.2%), 4 in HCS and 3 in HTK (p:0.551). Mean CPB time was 84min(42-110) and Cross-clamp time was 50min(24-65) with no difference between groups. Number of grafts performed were 3(1-5) Perioperative infarction occurred in 11(12%) patients, 5(7.9%) in HTK and 6(5.9%) in HCS (p:0.4), postoperative atrial fibrilation was present in 15(17.5%), 8 in HCS and 7 in HTK(p:0.4), Mean ICU time was 3days (1-5) for HTK and 5 for HCS (2-7). LOS was 4 for HTK and 6 days for HCS(p:0.2).

## Conclusion

CABG outcomes in high risk patients with an acute coronary syndrome was not affected by the type of cardioplegia.

